# Magnetic resonance imaging‐negative cerebral amyloid angiopathy: cerebrospinal fluid amyloid‐β_42_ over amyloid positron emission tomography

**DOI:** 10.1002/alz.087904

**Published:** 2025-01-09

**Authors:** Jung‐Min Pyun, Min Ju Kang, Seung Jin Baek, Kyungbok Lee, Young Ho Park, SangYun Kim

**Affiliations:** ^1^ Soonchunhyang University Seoul Hospital, Seoul Korea, Republic of (South); ^2^ Veterans Health Service Medical Center, Seoul Korea, Republic of (South); ^3^ Bobath Memorial Hospital, Seongnam‐si, Gyeonggi‐do Korea, Republic of (South); ^4^ Soonchunhyang University Seoul Hospital, Yongsan‐gu, Seoul Korea, Republic of (South); ^5^ Seoul National University College of Medicine & Neurocognitive Behavior Center, Seoul National University Bundang Hospital, Seoul Korea, Republic of (South); ^6^ Seoul National University College of Medicine & Seoul National University Bundang Hospital, Sungnam Korea, Republic of (South)

## Abstract

**Background:**

Cerebral amyloid angiopathy (CAA) pathology is becoming increasingly important in Alzheimer’s disease (AD) because of its potential link to amyloid‐related imaging abnormalities, a critical side effect observed during AD immunotherapy. Identification of CAA without typical magnetic resonance imaging (MRI) markers (MRI‐negative CAA) is challenging, and novel detection biomarkers are needed.

**Method:**

We included 69 participants with high neuritic plaques (NP) burden, with and without CAA pathology (NP with CAA vs. NP without CAA) based on autopsy data from the Alzheimer’s Disease Neuroimaging Initiative. Two participants with hemorrhagic CAA markers based on MRI were excluded and the final analysis involved 36 NP without CAA and 31 NP with CAA. A logistic regression model was used to compare the cerebrospinal fluid (CSF) amyloid‐β_42_ (Aβ_42_), phosphorylated tau_181_, and total tau levels, the amyloid positron emission tomography (PET) standardized uptake ratio (SUVR), and cognitive profiles between NP with and without CAA. Regression models for CSF and PET were adjusted for age at death, sex, and the last assessed clinical dementia rating sum of boxes score. Models for cognitive performances was adjusted for age at death, sex, and education level.

**Result:**

NP with CAA had significantly lower CSF Aβ_42_ levels when compared with those without CAA (110.5 pg/mL vs. 134.5 pg/mL, p‐value = 0.002). Logistic regression analysis revealed that low CSF Aβ_42_ levels were significantly associated with NP with CAA (odds ratio [OR]: 0.957, 95% confidence interval [CI]: 0.928, 0.987, p‐value = 0.005). However, amyloid PET SUVR did not differ between NP with CAA and those without CAA (1.39 vs. 1.48, p‐value = 0.666). Logistic regression model analysis did not reveal an association between amyloid PET SUVR and NP with CAA (OR: 0.360, 95% CI: 0.007, 1.741, p‐value = 0.606).

**Conclusion:**

CSF Aβ_42_ is more sensitive to predict MRI‐negative CAA in high NP burden than amyloid PET.

